# Dimensional convergence at the nanoscale: a novel synthesis of experimental evidence justifying direct measurement of quantum coherence in microtubules

**DOI:** 10.3389/fmolb.2026.1840116

**Published:** 2026-10-09

**Authors:** Justin Echternach

**Affiliations:** Independent Researcher, Tampa, FL, United States

**Keywords:** quantum coherence, microtubules, nanoconfinement, tryptophan networks, superradiance, quantum biology

## Abstract

We synthesize convergent experimental evidence from five independent research groups (2016–2025) addressing the longstanding “too warm, too wet” objection to quantum biology. Three recent experiments (2024–2025) demonstrate: (i) Wang et al. measured enhanced dielectric response and proton conductivity in nanoconfined water at 295 K, establishing extreme in-plane Coulomb screening at 1–2 nm; (ii) Rodríguez-Rubio et al. synthesized porphyrin nanobelts demonstrating room-temperature ring currents in 80–120 π-electron networks; (iii) Babcock et al. measured optical signatures consistent with ultraviolet superradiance from tryptophan networks in microtubules, with assembled microtubules showing enhanced fluorescence quantum yields relative to free tryptophan and tubulin dimers. Together with (iv) Reiter et al., who measured quantum-coherent proton motion in nanoconfined water via Compton scattering at 300 K, and (v) Larson et al., who demonstrated that microtubule structural integrity is functionally essential for computational coordination in unicellular *Euplotes*, these studies support a mechanistic chain from nanoconfinement through an aromatic quantum substrate and cooperative optical behavior to functional dependence. We argue this convergence justifies direct experimental measurement of decoherence times in microtubules as a necessary first step before addressing any functional role. We outline falsifiable predictions including dielectric mapping and anesthetic perturbation assays, and discuss implications for frameworks such as Orchestrated Objective Reduction. A direct link to consciousness is unproven; nonetheless, the data are consistent with quantum-coherent aromatic networks in biological scaffolds and motivate targeted falsification experiments.

## 1 Introduction

For nearly a century, biology was assumed to operate according to classical physics. Biological systems, being warm, wet, and noisy, seemed incompatible with quantum coherence. Tegmark’s 2000 calculation (Tegmark, 2000) argued that quantum coherence in microtubules would survive only ∼10^−13^ s, effectively dismissing quantum-consciousness theories such as Orchestrated Objective Reduction (Orch OR) (Hameroff and Penrose, 1996) as physically untenable.

Hagan et al. (2002) rebutted Tegmark, identifying several incorrect assumptions and recalculating decoherence times in microtubules at 10^−6^–10^−4^ s under more realistic conditions. Yet the “too warm, too wet” objection persisted for two decades. Early observations in photosynthetic complexes shifted the debate: Engel et al. (2007) reported coherent signatures in the Fenna–Matthews–Olson complex at 77 K, and Collini et al. (2010) reported similar signatures in marine algae at 294 K. These early interpretations have since been contested and partly reattributed to vibronic coherence (Section 3.6.1), but they nonetheless forced reconsideration of the thermal objection in at least some biological systems.

Three developments reopened the question for cytoskeletal structures. First, measurement precision improved: femtosecond spectroscopy, scanning dielectric microscopy, and single-molecule methods allow direct observation of fast and weak signals. Second, researchers began explicitly testing whether biological structures exploit quantum mechanics for functional advantage. Third, nanoconfinement emerged as a candidate protective mechanism: water confined on nanometer scales shows altered dielectric, structural, and transport properties. Biological nanoscale structures, including the ∼1–2 nm tryptophan nearest-neighbor separations and the associated hydrophobic pockets of the microtubule lattice, became plausible environments where quantum effects might be stabilized. Aromatic amino acids, particularly tryptophan, gained attention as potential quantum substrates.

Some authors have further proposed that biological systems may operate across multiple frequency domains, with short-lived high-frequency coherence coupling via phase relationships to slower, better-shielded collective modes (Hameroff et al., 2026). We treat that proposal as speculative and separable from the present argument; it is discussed only in the Supplementary Material. The core of this paper concerns the *spatial* convergence of five independent measurements on a critical 1–2 nm lengthscale, which provides a foundation for the direct tests proposed below.

Between 2016 and 2025, five independent research groups produced results that, although originally motivated by distinct questions, now co-localize on a common nanoscale picture:

- Nanoconfined water: Wang et al. (2025) used scanning dielectric microscopy to show that water confined to ∼1.5 nm channels on hexagonal boron nitride (hBN) surfaces exhibits an in-plane dielectric constant ε∥ ≈ 1,030 ± 350 and proton conductivity σ∥ ∼ 3 S m^-1^ at 295 K, implying ∼10× weaker static Coulomb coupling and extreme proton mobility in precisely the 1–2 nm regime.
- Quantum water precedent: Reiter et al. (2016) used Compton scattering to reveal quantum-coherent proton motion in water confined in carbon nanotubes of 1.4–1.6 nm diameter, with anomalously fast transport persisting to 300 K.
- Aromatic ring currents: Rodríguez-Rubio et al. (2025) synthesized triple-stranded porphyrin nanobelts with 80–120 π-electrons and diameters of 2–3 nm, showing strong global aromatic and antiaromatic ring currents at 298 K via nuclear magnetic resonance (NMR) and nucleus-independent chemical shift (NICS) analysis.
- Microtubule superradiance: Babcock et al. (2024) measured fluorescence quantum yields in tryptophan networks and found that intact microtubules exhibit enhanced quantum yields at 298 K relative to both free tryptophan and tubulin dimers, with large supramolecular assemblies exhibiting scaling consistent with Dicke-type superradiance.
- Functional microtubule (MT) computation: Larson et al. (2022) demonstrated that a unicellular ciliate, *Euplotes eurystomus*, uses microtubule-based fiber networks for gait coordination, with paclitaxel stabilization improving coordination and nocodazole-induced disruption abolishing it, indicating a structurally dependent computational role for microtubules that is conserved over >1 billion years.

These studies satisfy strong independence criteria: no shared authorship or institutional collaboration among the five primary experimental papers; five distinct techniques (dielectric microscopy, Compton scattering, NMR, fluorescence spectroscopy, and high-speed video with behavioral modeling); and a temporal span from 2016 to 2025. The 1–2 nm regime is also where the directly-measured anomalies *peak*: Wang’s dielectric enhancement maximizes near h ≈ 1.5 nm and decays toward bulk behavior above ∼4 nm, and Reiter’s quantum-coherent proton motion is specific to 1.4–1.6 nm confinement. The set is also heterogeneous in kind. In three cases (Wang, Reiter, Rodríguez-Rubio) the 1–2 nm value is a *measured* anomaly scale; in the biological cases (the tryptophan spacings Babcock probes, and the microtubule lattice geometry underlying Larson’s networks) it is a *structural* dimension that places the relevant geometry inside that same window, importing the nanoscale physics rather than independently re-measuring it. The claim is therefore one of scale co-localization (the microtubule lattice sits inside the window where the measured anomalies peak), not five independent rediscoveries of a single number. All key measurements are at or near physiological temperature (295–300 K).

### 1.1 Primary contribution

We report the first synthesis explicitly connecting these five independent studies as convergent evidence relevant to microtubule-relevant dimensional scales. No prior work has systematically emphasized the scale-matching principle: Reiter et al.‘s direct measurement of quantum coherence in water confined at 1.4–1.6 nm provides experimental precedent at the exact dimensional regime of the microtubule tryptophan nearest-neighbor spacings, where Wang’s dielectric anomaly and Babcock’s superradiance are most pertinent. We argue that this dimensional and mechanistic convergence justifies direct experimental investment in decoherence-time measurements in intact microtubules.

Two peer-reviewed 2026 reviews now survey quantum and collective optical effects in the cytoskeleton directly (Gassab and Craddock, 2026; Bilezikian et al., 2026), marking a shift from fringe speculation toward active mainstream inquiry. The present synthesis is distinct from these surveys: rather than cataloguing internal cytoskeletal evidence or shared-features reasoning, it identifies a cross-domain dimensional convergence at the 1–2 nm scale and argues that this convergence is what makes direct decoherence-time measurement the decisive next step. Both reviews, written independently of this framework, identify the central open question as whether the measured microtubule signatures require a collective or quantum description or can be accounted for by classical photophysics. That two disinterested surveys converge on this same unresolved question is not a weakness here; it is precisely the gap the experiments proposed in Section 5 are designed to close.

Section 2 outlines our analytical methodology. Section 3 analyzes each primary paper’s contribution to the mechanistic chain (Wang, Rodríguez-Rubio, Babcock, Reiter, Larson) and briefly reviews supporting evidence from photosynthesis, anesthesia, interfacial water, and microtubule electrodynamics. Section 4 discusses mechanistic connections, limitations, the “too warm, too wet” objection, and implications for consciousness frameworks. Section 5 summarizes convergent evidence and proposes specific, falsifiable experiments.

### 1.2 Scope and explicit limitations

This synthesis does not claim a direct link between quantum effects and consciousness. We distinguish clearly between what has been directly measured (Wang’s dielectric properties, Reiter’s quantum coherence in carbon nanotube water, Rodríguez-Rubio’s ring currents in porphyrins, Babcock’s fluorescence in microtubules, Larson’s microtubule-based coordination) and what remains plausible but untested (transferability of hBN-scale nanoconfined water behavior to protein interfaces, whether fluorescence definitively implies superradiance, whether paclitaxel’s enhancement reflects quantum vs. classical structural effects). Critical experimental tests remain open and are proposed in Section 5.

## 2 Methods: analytical approach

### 2.1 Paper selection criteria

We analyzed five experimental studies (2016–2025) selected on the basis of: (1) direct physical measurements rather than pure simulations; (2) room-temperature operation (295–310 K) or clear extrapolation to that range; and (3) independent research groups with no shared authorship or institutional collaboration. Wang et al. (2025) measured nanoconfined water properties using scanning dielectric microscopy. Rodríguez-Rubio et al. (2025) synthesized porphyrin nanobelts to probe aromatic ring currents. Babcock et al. (2024) measured fluorescence quantum yields in microtubule tryptophan networks. Reiter et al. (2016) probed water confined in carbon nanotubes via Compton scattering. Larson et al. (2022) demonstrated microtubule-based computational coordination in *Euplotes*.

The dimensional convergence at 1–2 nm emerged organically from the original experimental designs and was not selected *post hoc*.

### 2.2 Analytical framework: dimensional convergence

Our framework centers on dimensional convergence, understood as scale co-localization: a common lengthscale shared across independent measurements and structures, rather than five independent rediscoveries of a single value. The 1–2 nm range corresponds to fundamental structural features in proteins (e.g., side-chain separations, hydrophobic pockets), water ordering layers, and inter-aromatic (excitonic) separations. We distinguish two categories within the set: systems in which 1–2 nm is the *measured* scale of an anomaly (Wang’s dielectric peak, Reiter’s coherent-proton confinement, Rodríguez-Rubio’s ring-current cavities), and biological systems in which 1–2 nm is a *structural* dimension (the microtubule tryptophan nearest-neighbor spacings) that imports the same physics. The argument is that the microtubule lattice sits inside the scale window where the measured anomalies peak, not that all systems independently rediscover the scale.

We extracted dimensional parameters from each primary study. Wang et al. reported maximum dielectric enhancement at h ≈ 1.5 nm. Reiter et al. focused on carbon nanotube (CNT) inner diameters of 1.4–1.6 nm. Rodríguez-Rubio’s porphyrin nanobelts have cavity diameters of ∼2–3 nm. In microtubules the relevant 1–2 nm feature is the tryptophan nearest-neighbor spacing (∼1.3–1.8 nm, measured here from Protein Data Bank (PDB) entries 1JFF and 3JAK; Section 3.3.5), with the hydrophobic pockets near these residues and the structured interfacial-water layers adjacent to the protein wall as associated features on the ∼1 nm scale; this is distinct from the protofilament center-to-center spacing (∼5 nm), which is not the relevant scale. Dimensional convergence across methods and materials is treated as a primary organizing principle. An independent compilation reinforces this focus on the nanometer regime: Gassab and Craddock (2026) tabulate a diffusion-length hierarchy for the tubulin tryptophan system spanning sub-nanometer Dexter transfer, ∼1.5 nm Förster transfer, the ∼6.6 nm exciton migration measured by Kalra et al., and a longer coherent theoretical upper bound, placing the relevant transport physics at and just beyond the 1–2 nm scale identified here.

### 2.3 Mechanistic chain analysis

We then asked whether the five studies collectively address the minimal set of requirements for biologically relevant quantum coherence in microtubules:

1. A protective environment that modifies decoherence-relevant electromagnetic properties at the nanoscale (nanoconfined water).
2. A substrate capable of sustaining extended π-electron delocalization at room temperature (aromatic networks).
3. Empirical signatures of collective quantum behavior in a biological implementation (microtubule tryptophan networks).
4. Functional dependence of cellular computation on microtubule structural integrity (Larson’s finite-state machine).
5. Evolutionary depth and conservation, indicating that any such mechanism is not a late or isolated innovation.

We mapped each primary paper to one or more links in this chain and summarized them in Table 1; the dimensional convergence is plotted in Figure 1 (Section 2.3), and the mechanistic chain is developed in Section 4.1. Detailed quantitative results are presented once in Section 3; later sections refer back to those results rather than re-deriving them.

**TABLE 1:** Convergent evidence from five independent studies (2016–2025).

Study	System and method	Key finding	Scale/T	What’s measured	What’s inferred
Wang et al. (2025)	Water in hBN nanochannels; scanning dielectric microscopy	ε∥,hf ≈ 1,030 ± 350 and σ∥ ∼ 3 S m^-1^ at h ≈ 1.5 nm	1–2 nm; 295 K	ε∥, σ∥ on hBN	∼10× weaker Coulomb coupling; biological transferability untested
Reiter et al. (2016)	Water in CNTs; Compton scattering	Quantum-coherent proton motion; anomalous transport	1.4–1.6 nm; 20–300 K	Proton momentum distributions	Coherent motion of multiple protons; classical MD fails
Rodríguez-Rubio (2025)	Porphyrin nanobelts; ^1^H NMR, NICS	Global ring currents at 298 K in 80–120 π-electron belts	2.1–3.2 nm Ø; 298 K	Chemical shifts, ring-current susceptibilities	Room-temperature π-electron delocalization; scalability to larger networks
Babcock et al. (2024)	Tryptophan networks in MTs; fluorescence spectroscopy	Quantum-yield enhancement in intact MTs and mega-networks; scaling consistent with superradiance	∼1.3–1.8 nm tryptophan spacing; 298 K	Fluorescence quantum yields vs. network size	Cooperative optical effects; superradiant-like scaling
Larson et al. (2022)	*Euplotes* MT fiber networks; high-speed video, behavioral modeling	MT-based finite-state-machine-like coordination; paclitaxel enhances, nocodazole disrupts	∼1.3–1.8 nm tryptophan spacing; 295–300 K	Gait-state transitions, entropy production, mutual information	Structural MT integrity essential for computation; ancient conservation

**FIGURE 1:**
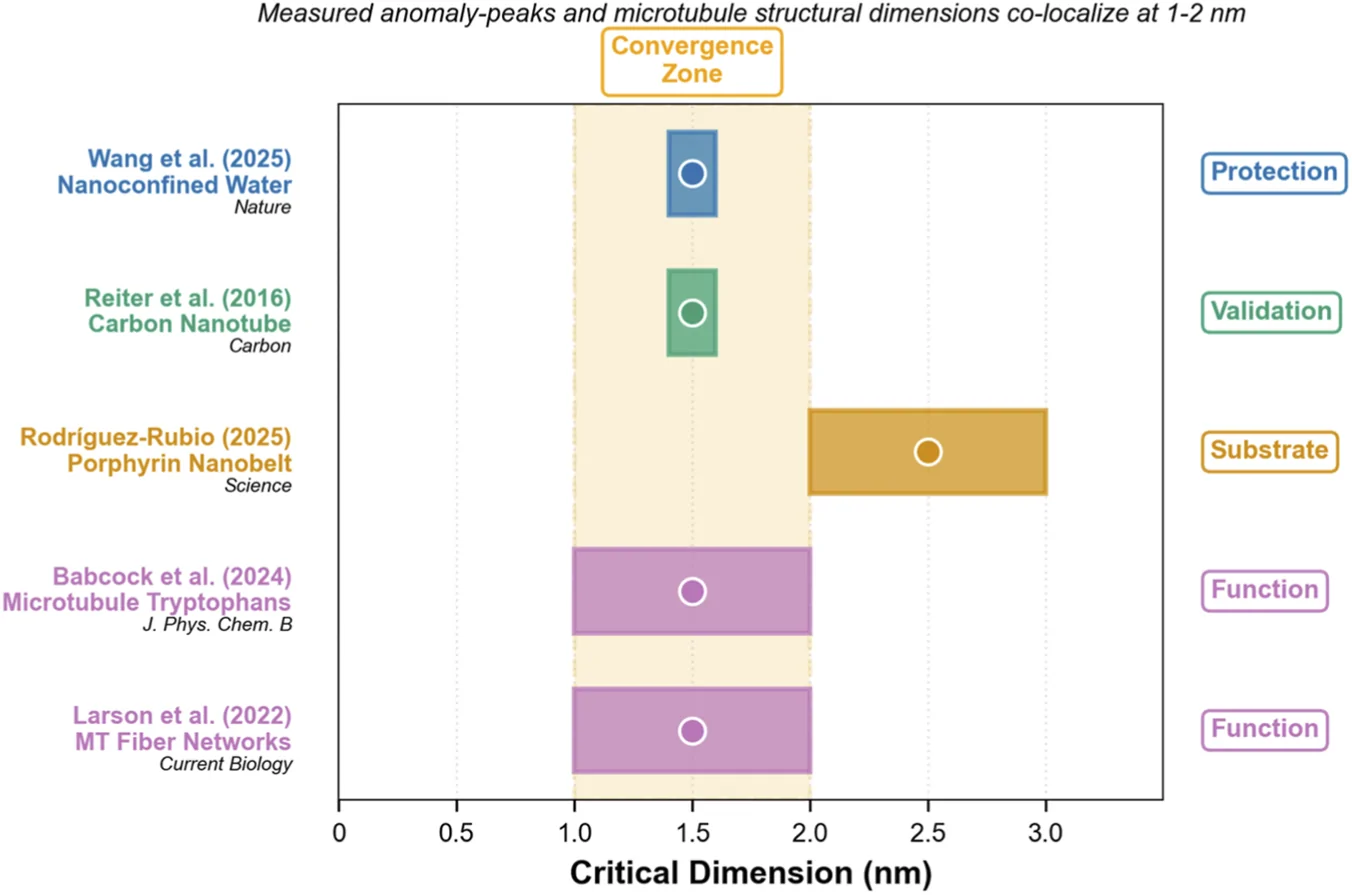
Dimensional convergence across five independent studies. Horizontal bars indicate the critical geometric lengthscale at which each system exhibits anomalous dielectric, transport, aromatic, optical, or functional behavior. Wang et al. (2025) – channel height where ε∥ and σ∥ peak for nanoconfined water (h ≈ 1.5 nm, blue); Reiter et al. (2016) – inner diameter of CNTs showing quantum-coherent proton motion (d = 1.4–1.6 nm, green); Rodríguez-Rubio et al. (2025) – inner diameter of porphyrin nanobelts exhibiting global ring currents (≈ 2–3 nm, orange); Babcock et al. (2024) – tryptophan nearest-neighbor separations and hydrophobic pockets (∼1.3–1.8 nm, purple) in the microtubule lattice, underlying the tryptophan networks whose quantum yield is measured; Larson et al. (2022) – microtubule fiber networks built from that same lattice (purple). The gold shaded region marks the 1–2 nm “convergence zone,” corresponding to the tryptophan nearest-neighbor spacings (∼1.3–1.8 nm) and the structured interfacial-water layers of the microtubule lattice, not the center-to-center protofilament spacing. Only some of these studies directly resolve the 1–2 nm confinement; for the biological systems, the plotted values refer to the known structural features that import this nanoscale physics into the larger assemblies.

### 2.4 Structural distance measurement

Tryptophan separations were measured directly from deposited atomic coordinates using custom Python scripts (with NumPy for coordinate geometry), applied to PDB entries 1JFF (the αβ-tubulin heterodimer) and 3JAK (the assembled microtubule lattice). For each conserved tryptophan, the indole centroid was defined as the geometric center of the nine non-hydrogen atoms of the indole ring system (CG, CD1, CD2, NE1, CE2, CE3, CZ2, CZ3, CH2). For every α/β tryptophan pair, both within the heterodimer (1JFF) and across the inter-dimer boundary in the lattice (3JAK), we computed the centroid–centroid separation and the minimum interatomic (closest ring-atom) distance. The resulting distances are reported in Section 3.3.5. Inter-protofilament geometry was measured from the same lattice (3JAK): the protofilament center-to-center spacing from laterally adjacent tubulin centroids, and the closest inter-protofilament contact from minimum interatomic distances. Scripts are available from the author on request.

## 3 Results: three primary studies and two bridges

### 3.1 Nanoconfined water exhibits extreme dielectric properties

#### 3.1.1 Experimental design

Wang et al. (2025) employed scanning dielectric microscopy (SDM) to measure the in-plane dielectric constant (ε∥) and conductivity (σ∥) of water confined in hexagonal boron nitride (hBN) nanochannels as a function of confinement height h at 295 K. Van der Waals–assembled channels formed between hBN and graphite allowed controllable confinement heights from h ≈ 1 nm up to tens of nanometers. All measurements reported in that study were performed at room temperature (295 K) (Wang et al., 2025; Fumagalli et al., 2012; Fumagalli et al., 2018). The relevance of this in-plane field geometry for biological nanoconfinement at protein interfaces and microtubule gaps is discussed in Section 3.1.4.

#### 3.1.2 In-plane dielectric constant

At h > 4 nm, water exhibits bulk-like ε∥,hf ≈ 74 ± 17. At the critical dimension h ≈ 1–2 nm, the dielectric constant reaches ε∥,hf ≈ 1,030 ± 350, a ∼13-fold enhancement over bulk water (Figure 2). This ferroelectric-range permittivity arises from confinement-induced ordering and correlated dipole reorientations.

**FIGURE 2:**
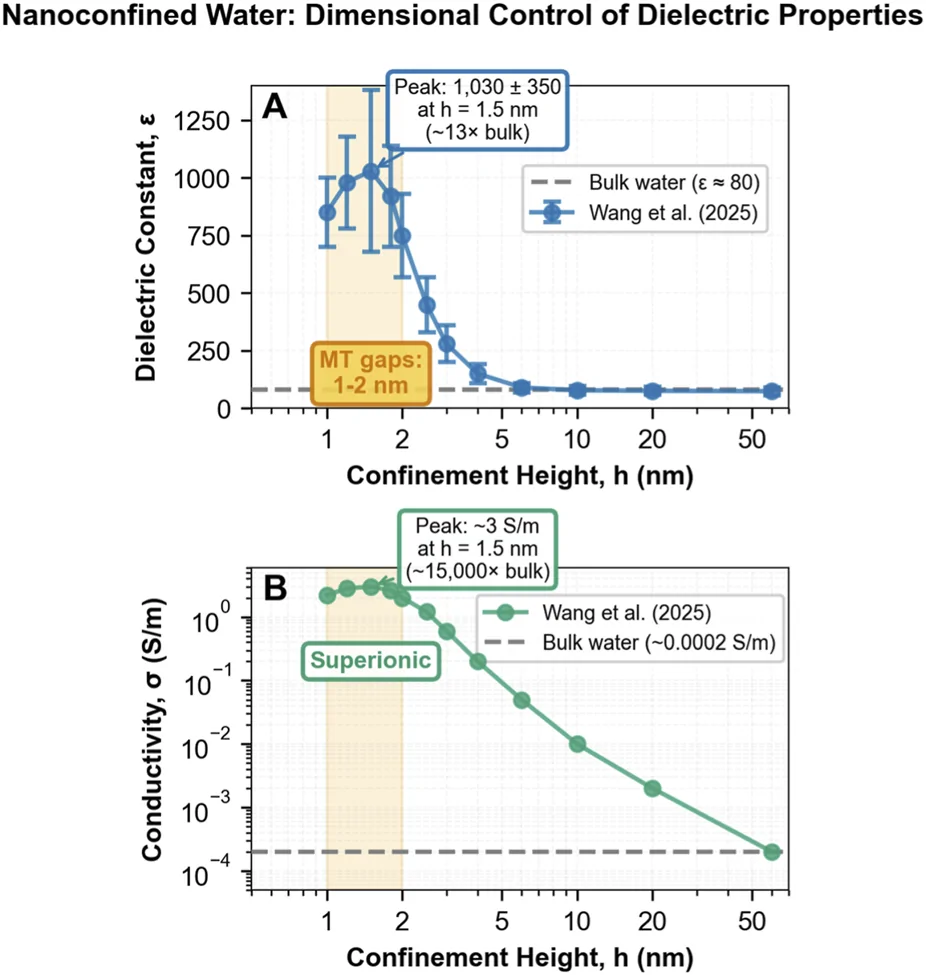
Nanoconfined water dielectric properties from Wang et al. (2025). **(A)** In-plane high-frequency dielectric constant (ε∥) vs. confinement height (h) shows a peak at h ≈ 1.5 nm with ε∥ ≈ 1,030 ± 350 (∼13× bulk). **(B)** In-plane proton conductivity (σ∥) vs. h shows a peak near h ≈ 1.5 nm at σ∥ ≈ 3 S/m (∼10^4^–10^5^× bulk). Gold band highlights the 1–2 nm regime that matches microtubule tryptophan nearest-neighbor spacings (∼1.3–1.8 nm) and protofilament-scale interfacial-water regions.

For microtubule-relevant geometries, an order-of-magnitude increase in ε∥ implies a corresponding reduction in static Coulomb coupling between charges, suggesting that nanoconfined water may act as an electrostatic shield that could, in principle, extend coherence times for electronic excitations in nearby aromatic networks (Section 4.1).

#### 3.1.3 In-plane proton conductivity

At h ≈ 1.5 nm, conductivity reaches σ∥ ∼ 3 S m^-1^, roughly 10^4^–10^5^ times higher than bulk water (σ_bulk ≈2 × 10^−4^ S/m). This enhancement reflects efficient proton transport via the Grotthuss mechanism (Marx et al., 1999) in a disrupted hydrogen-bond network. For the microtubule tryptophan microenvironment and wall-adjacent water layers at this scale, this suggests that strongly conducting protonic layers could coexist with high dielectric screening.

#### 3.1.4 Critical dimension and biological relevance

The critical dimension h ≈ 1.5–2 nm coincides with the microtubule tryptophan nearest-neighbor spacings (∼1.3–1.8 nm; Section 3.3.5) and the hydrophobic pockets near those residues in tubulin dimers (Nogales et al., 1998; Löwe et al., 2001), not the ∼5 nm protofilament center-to-center spacing. If Wang’s measurements transfer qualitatively to protein surfaces, water confined in these pockets and in the wall-adjacent layers could exhibit ε∥ on the order of several hundred to ∼1,000 and σ∥ in the multi-S/m range. This would reduce Coulomb interactions, modify local noise spectra, and potentially influence decoherence times for excitonic or ring-current states in nearby aromatic networks.

#### 3.1.5 Mechanistic implications

The decoherence rate due to electrostatic noise scales approximately as Γ ∝ 1/ε^2^ for static contributions: the rate varies as the square of the dielectrically screened Coulomb coupling, so enhanced screening lengthens the decoherence time (Tegmark’s decoherence model (Tegmark, 2000); the screening-based reduction of microtubule decoherence set out by Hagan et al. (2002). The observed ε∥ enhancement at 1–2 nm scales could reduce static components of decoherence by an order of magnitude or more. Whether dynamic fluctuations are also suppressed remains unknown: superionic proton conduction (the σ∥ ∼ 3 S m^-1^ measured by Wang et al. (2025), carried by a Grotthuss-type proton-hopping mechanism (Marx et al., 1999) might introduce fast charge fluctuations, but timescale separation (proton hopping on ps–ns vs. electronic coherence on fs–ps) could mitigate deleterious effects. Resolving this requires direct decoherence-time measurements in microtubule aromatic networks (Section 5).

Taken together, Wang’s measurements and the structural dimensions of microtubules identify a concrete physical locus for environmental protection: the structured, nanoconfined water at the tryptophan-bearing surfaces and narrow crevices of the MT lattice. In the remainder of this manuscript we treat this nanoconfined aqueous layer as the candidate “filtering medium” that can both suppress static Coulomb interactions from the cytosol and support the coherent electronic dynamics inferred from the aromatic network (Sections 3.6 and 5).

### 3.2 Porphyrin nanobelts sustain global ring currents

#### 3.2.1 Experimental design

Rodríguez-Rubio et al. (2025) synthesized triple-stranded edge-fused porphyrin nanobelts (cf-PN, N = 8–12) with diameters of 2.1–3.2 nm containing 80–120 π-electrons. Triple connectivity enhances electronic coupling; edge-fusion enforces near planarity and uninterrupted π-electron flow. The alternation between 4n and 4n+2 electron counts tests whether Hückel’s rule (Hückel, 1931), originally derived for benzene, extends to nanometer-scale aromatic circuits (Table 2).

**TABLE 2:** Porphyrin Nanobelt Quantum Properties (Rodríguez-Rubio et al., 2025).

Nanobelt	N	π-electrons	Hückel class	NICS(0)zz	χ (nA/T)	Character
cf-P8	8	80 (4n)	Antiaromatic	+34.9	+41.9	Strong paratropic
cf-P9	9	90 (4n+2)	Aromatic	−19.1	−29.8	Strong diatropic
cf-P10	10	100 (4n)	Antiaromatic	+17.5	+31.3	Moderate paratropic
cf-P11	11	110 (4n+2)	Aromatic	−10.6	−20.0	Moderate diatropic

Primary characterization used ^1^H NMR spectroscopy and computed NICS(0)zz values (Schleyer et al., 1996) at 298 K, probing ring currents via shielding/deshielding patterns.

#### 3.2.2 Room-temperature global ring currents

NMR data demonstrate global quantum delocalization consistent with Hückel’s rule:

- Odd-numbered belts (N = 9, 11; 4n+2 π-electrons) exhibit aromatic diatropic currents: inner methyl protons are strongly shielded (upfield shifts), outer protons deshielded.
- Even-numbered belts (N = 8, 10, 12; 4n π-electrons) exhibit antiaromatic paratropic currents with reversed patterns.

Ring current susceptibilities reach |χ| ≈ 30–40 nA/T, 2.5–3.5× stronger than benzene despite spanning 2–3 nm circumferences and carrying 80–120 π-electrons.

All measurements were made at 298 K, demonstrating that large aromatic π-electron networks can sustain extended quantum delocalization under ambient thermal conditions.

#### 3.2.3 Biological relevance

The porphyrin nanobelts provide a scaling bridge:

- Benzene (6 π-electrons) → tryptophan (10 π-electrons),
- Chlorophyll/heme (18 π-electrons, known to support quantum coherence in photosynthesis and respiration),
- To synthetic porphyrin belts (80–120 π-electrons at 2–3 nm),
- And finally to microtubule tryptophan networks (>10^5^ π-electrons in extended arrays).

If Hückel-type selection rules and ring-current behavior apply to 120 π-electron systems at 298 K, it is plausible that much larger, structured aromatic networks in microtubules can support coherent excitonic or current states, provided geometrical constraints (stacking distances, connectivity) are met. Babcock et al.‘s measurements (Section 3.3) directly probe such networks in a biological context.

### 3.3 Superradiance in microtubule tryptophan networks

Babcock et al. (2024) analyze ultraviolet (UV) emission from large networks of tryptophan (Trp) embedded in tubulin architectures. Their work combines experimental fluorescence quantum yield (QY) measurements on simple assemblies with theoretical modeling of radiative coupling in larger, biologically realistic structures (centrioles and axonal microtubule bundles). This section clearly separates those two roles.

#### 3.3.1 Experimental QY measurements: Trp → TuD → MTs

The experimentally measured part of the study focuses on three systems:

- Free tryptophan in solution (baseline),
- tubulin dimers (TuD, 8 Trp/dimer),
- assembled microtubules (∼13,000 Trp per 1 μm MT).

All measurements are performed at 298 K in aqueous buffer, with excitation at 280 nm and emission collected in the 300–400 nm range.

The reported QY values show a non-trivial, size-dependent trend:

- Free Trp: QY ≈ 12.4 ± 1.1% (baseline).
- Tubulin dimer: QY ≈ 6.8 ± 0.4% (quenched relative to free Trp).
- Assembled MTs: QY ≈ 17.6 ± 2.1% (mean, range 12.7%–21.3%).

Two key points follow from these measurements:

1. Assembly matters: isolated Trp is moderately bright; Trp embedded in the tubulin dimer is quenched; but when dimers assemble into microtubules, the QY not only recovers but exceeds the free-Trp baseline. This suggests that higher-order organization can relieve local quenching and enable more efficient radiative pathways.
2. Cooperative behavior is implicated: the QY enhancement in assembled MTs relative to dimers (+160% over TuD and +42% over free Trp, on average) is difficult to explain by purely local, independent-emitter models. It motivates an interpretation in terms of collective excitonic states and cooperative emission, i.e., superradiance-like behavior.

No direct QY measurements are reported for centrioles or axonal microtubule bundles. Those larger systems enter the analysis only through theoretical modeling, described next.

#### 3.3.2 Dicke superradiance and theoretical enhancement in larger architectures

In Dicke superradiance (Dicke, 1954), N identical chromophores confined within a volume smaller than ${\left(\lambda /2\pi \right)}^{3}$ can behave as a single, collectively coupled quantum emitter. In the ideal case, the intensity scales as $I\propto N^{2}$ , and the radiative decay rate of the fully symmetric “bright” state scales as Γ∝N , while the majority of states become subradiant.

For Trp emission near 330 nm, the corresponding coherence radius is on the order of tens of nanometers, so segments of a microtubule (25 nm diameter) and closely packed higher-order assemblies can, in principle, support cooperative emission modes in the Dicke sense. However, real biological structures are extended, disordered, and anisotropic, so one expects deviations from simple $N^{2}$ scaling.

Babcock et al. explicitly address this by constructing an effective non-Hermitian excitonic Hamiltonian for larger Trp networks representing:

- Centrioles: nested barrel-like arrays of microtubule triplets.
- Axonal microtubule bundles: hexagonally packed MTs with realistic spacing and bundle diameters.

From this model they compute:

- The excitonic eigenstates and their radiative decay rates $\Gamma_{j}$ ,
- A superradiant enhancement factor $\max\left(\Gamma_{j}/\gamma \right)$ , where γ is the single-Trp decay rate,
- The dependence of this enhancement on system size and static energetic disorder.

Several points are crucial for an accurate synthesis:

- The dramatic enhancement factors quoted for centrioles (order $10^{3}$ – $10^{4}$ relative to dimers) and axonal bundles (approaching ∼7,000× ) refer to theoretical superradiant enhancement factors, not directly measured QY values.
- The scaling of $\max\left(\Gamma_{j}/\gamma \right)$ with system size is superlinear but saturating, rather than strictly N^2^ as in the ideal Dicke model. Realistic geometry and disorder redistribute oscillator strength among several bright modes and impose a finite saturation scale.
- Nonetheless, the enhancement remains large and robust at room temperature, even when static disorder of order $k_{B}T$ is included.

Babcock et al. thus provide a Dicke-inspired but biologically realistic picture: extended Trp networks in microtubule-based architectures can host superradiant-like modes with strong radiative enhancement, but the scaling is tempered by geometry and disorder. To keep the measured and modeled results clearly separated: the directly *measured* effect is a modest ∼1.4-fold quantum-yield increase in assembled microtubules over free tryptophan (17.6% vs 12.4%), whereas the $10^{3}$ – $10^{4}$ enhancement factors are *theoretical* predictions for centrioles and axonal bundles that have not been measured. Our argument uses the former as empirical evidence of cooperative behavior and treats the latter strictly as a motivating, testable prediction, not an established result.

#### 3.3.3 Implications for dimensional convergence and protected excitonic substrates

For the purposes of this dimensional convergence synthesis, Babcock et al. contribute three key elements:

1. Identification of a specific quantum substrate. Tryptophan networks embedded in tubulin and microtubule architectures are shown, both experimentally (QY trends for Trp → TuD → MTs) and theoretically (mode analysis in centrioles and bundles), to behave as extended excitonic systems capable of cooperative radiative behavior.
2. Link to Dicke-type collective emission without overclaiming. The framework explicitly references Dicke superradiance (Dicke, 1954) as the idealized limit, while demonstrating that realistic microtubule architectures can still realize large superradiant enhancement factors with superlinear, saturating scaling, rather than exact $N^{2}$ . This allows the manuscript to invoke Dicke’s paradigm without erroneously claiming that biological structures implement a perfect Dicke ensemble.
3. Room-temperature robustness in realistic geometries. The modeled superradiant modes remain bright and robust under static disorder comparable to $k_{B}T$ at 298 K. Combined with the dielectric protection afforded by nanoconfined water (Section 3.1), this supports the idea that microtubule architectures can host protected, room-temperature excitonic substrates suitable for sustained quantum coherence.

In the broader context of the manuscript’s mechanistic chain—nanoconfinement protection (Wang et al.), excitonic substrate and cooperative emission (Babcock et al.), electrical and quantum-device behavior (scanning tunneling microscopy [STM] and atomic force microscopy [AFM] work), and functional computation (Larson et al.)—Babcock et al. provide the crucial bridge between geometric architecture and cooperative UV excitonics in microtubule-based structures.

#### 3.3.4 Disorder robustness and “too warm” objection

Babcock et al. simulated disorder in their theoretical analysis and showed that superradiant behavior in microtubule-scale networks remains robust up to disorder strengths of ∼1000 cm^-1^, corresponding to ∼5 × kT at 298 K. This far exceeds typical disorder tolerances in photosynthetic complexes (100–300 cm^-1^) and directly addresses the “too warm” objection in the context of extended aromatic networks. Larger networks, counterintuitively, become more robust due to cooperative averaging of local perturbations.

#### 3.3.5 Tryptophan network architecture and anesthetic binding

Tubulin heterodimers contain eight conserved tryptophan residues (α: W21, W346, W388, W407; β: W21, W103, W346, W407) clustered at inter-dimer and inter-protofilament interfaces (Nogales et al., 1998; Löwe et al., 2001). We measured their separations directly from deposited coordinates. In the αβ-dimer (PDB 1JFF) the closest indole-centroid separations are 1.5–1.8 nm, with the αW407–βW346 pair at 1.53 nm (closest ring-atom approach 1.1 nm); in the assembled microtubule lattice (PDB 3JAK (Zhang et al., 2015)) the network continues across the inter-dimer boundary at 1.36–1.57 nm (αW346–βW407 and βW346–αW407). The tryptophan network is therefore continuous along the protofilament with nearest-neighbor spacings of ∼1.3–1.8 nm, within the near-field/excitonic dipole–dipole coupling range. The closest any two indole rings approach (1.1 nm) is far beyond the ∼3.5 Å of contact π–π stacking, so the network couples through transition-dipole (excitonic) interactions rather than contact π-stacking. The resulting network scales from ∼80 π-electrons per dimer (eight indole rings) to ∼10^5^–10^6^ π-electrons per microtubule or bundle. Measured from the same lattice (PDB 3JAK), adjacent protofilaments are separated by ∼5.4 nm center-to-center and make direct lateral contact at the M-loop interface (closest heavy-atom approach ∼3 Å); the ∼1–2 nm scale relevant here is therefore the tryptophan microenvironment (the nearest-neighbor spacings and associated pockets), not a uniform protofilament separation.

Craddock et al. (2015) identified these same hydrophobic pockets as preferred binding sites for diverse general anesthetics (isoflurane, propofol, xenon). In molecular-dynamics simulations, anesthetics binding in these pockets perturb the local aromatic geometry by ∼0.5–1.0 Å (Craddock et al., 2015); this figure is a perturbation magnitude (a binding-induced displacement), not the anesthetic-to-tryptophan distance, which is several ångströms across the hydrophobic pocket. The coincidence between anesthetic binding sites and superradiant networks motivates a specific and testable hypothesis: clinically relevant anesthetics should attenuate or disrupt the fluorescence enhancements associated with superradiant-like behavior in microtubules (Section 4.4 and 4.5.3).

### 3.4 Quantum coherence in nanoconfined water via Compton scattering

Reiter et al. (2016) used X-ray and neutron Compton scattering to probe proton momentum distributions in water confined within carbon nanotubes of 1.4–1.6 nm inner diameter. They observed features inconsistent with a classical ensemble of independent water molecules and interpreted their data as evidence for coherent motion of multiple protons persisting up to 300 K. Transport measurements in such nanotubes show water flow up to ∼60× faster than early classical predictions (Hummer et al., 2001; Striolo, 2006), consistent with quantum-enhanced transport.

Reiter’s study provides an independent, momentum-space confirmation that nanoconfined water at 1.4–1.6 nm behaves in a qualitatively quantum manner at room temperature. Although the material system (CNTs) differs from Wang’s hBN channels, both identify the same lengthscale and temperature regime for anomalous behavior. Together, Wang and Reiter support the notion that water in geometries matching the microtubule tryptophan nearest-neighbor spacings belongs to a distinct physical phase with modified electromagnetic properties and quantum transport characteristics.

### 3.5 Microtubule-based computational coordination in unicellular organisms

Larson et al. (2022) provide functional context by studying gait control in the unicellular ciliate *Euplotes eurystomus*, which walks using 14 cirri (bundled cilia). Microtubule-based kinetodesmal fibers connect cirri via a transverse microtubule ribbon, forming a structured fiber network.

#### 3.5.1 Experimental design

High-speed video (500–1000 fps) captured gait patterns, and Markov modeling identified 32 discrete gait states with non-random transition structure. Entropy production and mutual information between cirri pairs quantified non-equilibrium computational behavior.

#### 3.5.2 Pharmacological perturbation

Two interventions probed the role of microtubules:

- Paclitaxel (stabilizer, ∼10 μM): Improved coordination, straighter trajectories, reduced step variability, and increased mutual information between connected cirri. Transitions between gait states became more deterministic, indicating enhanced information throughput.
- Nocodazole (depolymerizer, 5–10 μM): Disrupted coordination; entropy production dropped from ∼0.4 nats to ∼0.1 nats per cycle, approaching near-equilibrium behavior. Mutual information between cirri collapsed towards baseline.

Laser microsurgery severing individual kinetodesmal fibers recapitulated local deficits, confirming that the microtubule fiber network is the information-carrying substrate rather than merely passive structure.

#### 3.5.3 Evolutionary and conceptual relevance

*Euplotes* diverged from the animal lineage over a billion years ago. The observation that microtubule-based fiber networks implement a finite-state-machine-like controller for gait, and that structural stabilization (paclitaxel) improves performance while depolymerization (nocodazole) undermines it, shows that microtubule-dependent computation is an ancient and strongly conserved cellular strategy.

Larson et al. do not measure quantum coherence, and their result is a classical one: microtubule structural order is functionally rate-limiting for cellular computation. We include it not as evidence for quantum coherence, but because it establishes that microtubule architecture is computationally load-bearing in a living cell, precisely the finding that motivates interrogating that architecture for quantum effects. The structural prerequisites it identifies (ordered lattice, stable geometry, preserved network connectivity) overlap those inferred from Wang, Rodríguez-Rubio, and Babcock, but that overlap is a reason to test the quantum hypothesis, not a confirmation of it.

### 3.6 Supporting evidence: photosynthesis, anesthesia, interfacial water, and microtubule electrodynamics

#### 3.6.1 Photosynthetic quantum coherence

Engel et al. (2007) and Collini et al. (2010) reported quantum-coherent signatures in chlorophyll networks in the FMO complex and marine algae at cryogenic and ambient temperature, respectively. The interpretation of these early observations has since been actively contested: much of the long-lived signal has been reattributed to vibrational (vibronic) rather than purely electronic coherence, and the functional role of any electronic coherence in photosynthesis remains debated. Recent full-microscopic simulations, however, recover persistent quantum effects in primary photosynthesis that coarser-grained vibrational models had obscured (Lorenzoni et al., 2025). We therefore invoke photosynthesis not as settled proof of functional biological coherence, but as the precedent that established room-temperature quantum effects in aromatic biological networks as a serious empirical question rather than an *a priori* impossibility.

#### 3.6.2 Anesthetic binding to aromatic sites

Craddock et al. (2015) used molecular dynamics simulations to show that chemically diverse anesthetics preferentially bind to hydrophobic pockets in tubulin containing tryptophan residues. These binding sites spatially overlap the aromatic networks probed by Babcock et al. (2024). Classical receptor-based models of anesthesia (e.g., GABA_A or NMDA receptor modulation (Franks and Lieb, 1994; Hemmings et al., 2005; Campagna et al., 2003)) remain important, but the alignment of anesthetic binding with quantum-active aromatic networks yields a distinct, testable prediction: anesthetics should perturb collective optical signatures in microtubules (Section 4.4, 4.5.3).

#### 3.6.3 Protein interfacial water

Protein hydration studies (Ball, 2008; Chaplin, 2006; Bellissent-Funel et al., 2016) show that water within ∼0.5–1 nm of protein surfaces exhibits altered mobility, structure, and dielectric behavior compared to bulk water, with effective dielectric constants often significantly reduced. While no current study measures ε∥ ≈ 1,000 at protein interfaces, the general principle that nanoscale confinement produces qualitatively new water phases is well supported. Wang’s nanoconfined water should therefore be regarded as a physical mechanism likely to generalize under certain geometric and chemical conditions, not an hBN-specific anomaly.

Independent support for the functional importance of structured water comes from Bilezikian et al. (2026), who argue that nanoconfined water may be not merely protective but, in some cytoskeletal contexts, strictly necessary for the relevant electronic effects, and who describe a glassy, amorphous-ice-like hydration phase forming homochiral domains with icosahedral local order, qualitatively distinct from bulk water in precisely the way Wang’s measurements imply.

#### 3.6.4 Microtubule electrodynamics (Sahu and Saxena)

Sahu et al. (2013) and Saxena et al. (2020), Saxena et al. (2023) provide the kind of biological corroboration one would expect if Wang’s nanoconfined-water physics applies inside the microtubule lattice.

Several studies on microtubule electrodynamics provide additional, albeit indirect, support:

- (Sahu et al., 2013) reported very high low-frequency dielectric constants and water-dependent conductance in microtubule solutions, with conductance dropping upon dehydration and recovering with rehydration, suggesting that structured water in the tubulin lattice is essential for electrodynamic behavior.
- (Saxena et al., 2020; Saxena et al., 2023) measured AC conductance and low-temperature transport in taxol-stabilized microtubules, observing resonant frequency peaks and signatures consistent with variable-range hopping, Coulomb blockade, and quantum interference. Resonances were weakly dependent on microtubule length, hinting at scale-free or topological collective modes.

When this structured interfacial and inter-protofilament water is removed or disrupted (for example, by drying or cooling in vacuum), these enhanced electrodynamic properties collapse and microtubules revert toward more insulating behaviour, exactly what one would expect if the nanoconfined water is the functional realization of the dielectric-protection mechanism identified by Wang et al. (2025). In this sense, Wang supplies the protective physics, while Sahu, Saxena and STM/AFM studies suggest that this protected 1–2 nm water phase is mechanistically important for MT conductance, switching and memory-like behaviour, consistent with their proposed role as mesoscopic quantum devices. These electrodynamic results come largely from a single group, however, and await independent replication; we therefore treat them as corroborative rather than decisive.

## 4 Discussion: mechanistic convergence and implications

### 4.1 The mechanistic chain: from nanoconfinement to function

Taken together, the five primary studies establish a set of necessary conditions for microtubule quantum coherence, which we summarize as a mechanistic chain (Figure 3). We stress that meeting these conditions is necessary, not sufficient: the chain identifies what must hold for quantum coherence to be possible and where the geometry and physics co-localize, not a derivation that coherence occurs.

1. Protection mechanism (nanoconfined water + electrodynamics):
  ○ Wang et al. (2025) show that water confined at 1–2 nm exhibits an anomalously large in-plane high-frequency dielectric constant (ε∥,hf ≈ 1,030 ± 350) at h ≈ 1.5 nm, implying an order-of-magnitude reduction in static Coulomb coupling compared to bulk water.
  ○ Reiter et al. (2016) independently observe quantum-coherent proton motion in water confined in 1.4–1.6 nm CNTs at 300 K, reinforcing that this dielectric enhancement is a robust feature of 1–2 nm aqueous confinement.
  ○ Sahu et al. (2013), Saxena et al. (2020), Saxena et al. (2023), together with STM/AFM studies of brain microtubules, demonstrate that structured microtubule water is mechanistically required for MT conductance and memory-like switching: drying or disrupting it collapses the enhanced electrodynamic behaviour. The protected, structured water phase is best understood as the interfacial shell adjacent to the protofilament wall and within the narrow inter-protofilament crevices, not the full ∼15 nm lumen, whose central region is expected to remain comparatively bulk-like; the nanoconfinement physics applies to the wall-adjacent and crevice water, with the lumen as a larger aqueous cavity.

**FIGURE 3:**
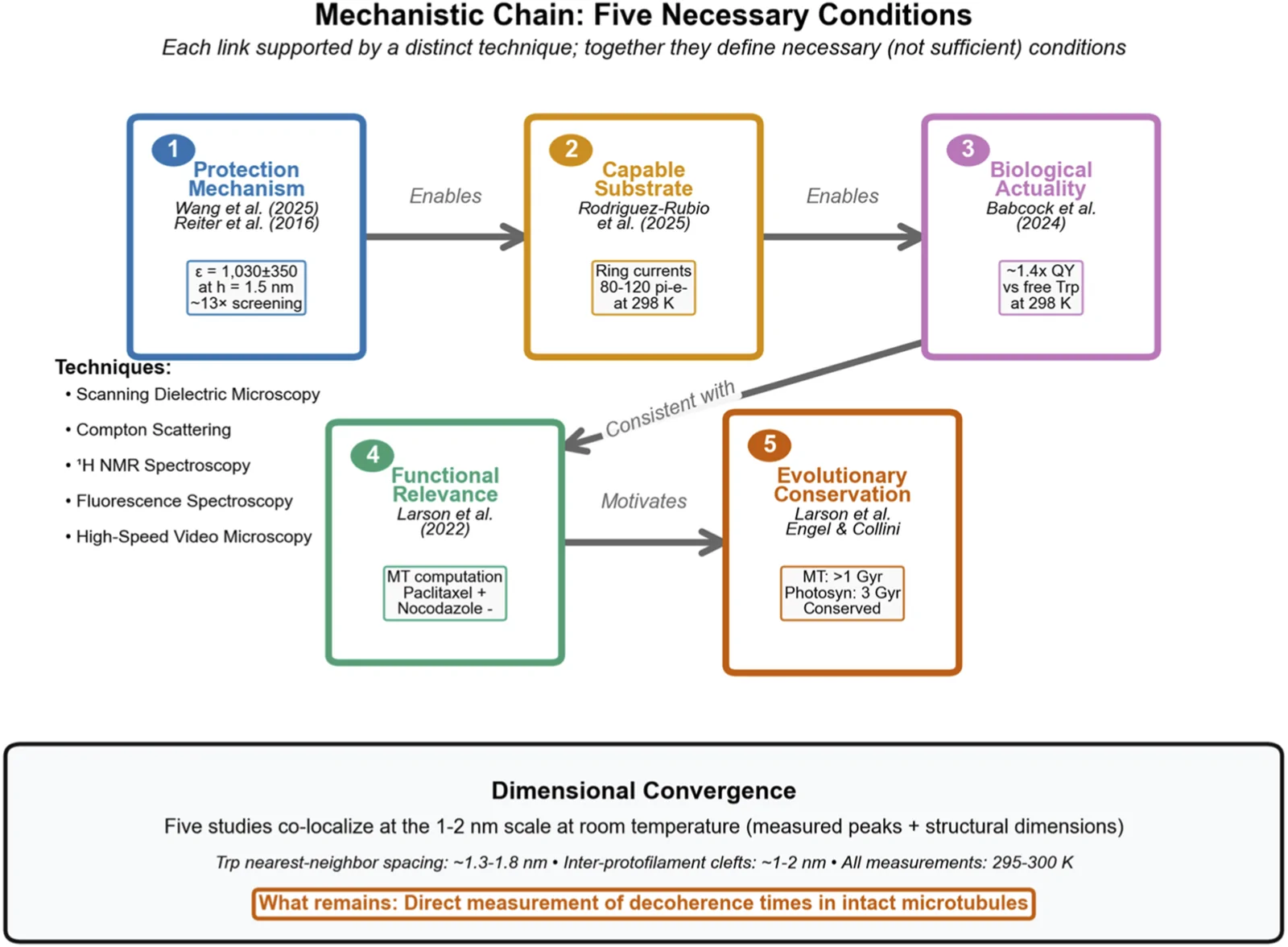
Complete mechanistic chain with five independent validations. Flowchart shows five sequential links, each independently verified by distinct experimental techniques: (1) Protection mechanism (Wang et al., 2025, Reiter et al., 2016) - nanoconfinement provides ∼13× Coulomb screening at h = 1.5 nm; (2) Capable substrate (Rodríguez-Rubio et al., 2025) - aromatic π-systems sustain ring currents at 298 K in 80–120 electron systems; (3) Biological actuality (Babcock et al., 2024) - quantum-yield enhancement (measured directly at 298 K; ∼1.4× over free tryptophan, ∼2.6× over the tubulin dimer), consistent with superradiance; (4) Functional relevance (Larson et al., 2022) - MT structural integrity enables computational coordination, enhanced by paclitaxel and disrupted by nocodazole; (5) Evolutionary conservation (Larson et al., 2022, Engel/Collini) - MT-based computation conserved >1 billion years, quantum biology conserved 1–3 billion years. Bottom panel emphasizes dimensional convergence: the five studies co-localize at the 1–2 nm scale at room temperature (295–300 K), combining measured peaks and structural dimensions; the porphyrin-nanobelt bridge extends to approximately 2–3 nm. Left legend lists experimental techniques: scanning dielectric microscopy, Compton scattering, ^1^H NMR spectroscopy, fluorescence spectroscopy, and high-speed video microscopy. Box at bottom highlights what remains unmeasured: direct decoherence time measurements in intact microtubules.

Taken together, these results suggest that the same 1–2 nm nanoconfined water layer that protects quantum states in Wang’s experiment is also the functional control element for microtubule electrical behaviour, providing both environmental protection and biological necessity.

2. Capable substrate (aromatic π-networks):
  ○ Rodríguez-Rubio et al. (2025) demonstrate that 80–120 π-electron porphyrin nanobelts support strong global ring currents at 298 K, extending Hückel-type aromaticity to nanometer-scale circuits.
3. Biological actuality (microtubule networks):
  ○ Babcock et al. (2024) measure size-dependent fluorescence enhancements in microtubule tryptophan networks and larger assemblies at 298 K, with scaling consistent with superradiance and robustness to disorder several times larger than kT.
4. Functional relevance (cellular computation):
  ○ Larson et al. (2022) show that microtubule structural integrity is functionally critical for finite-state-machine-like coordination in *Euplotes*, with stabilization (paclitaxel) enhancing performance and depolymerization (nocodazole) abolishing it.
5. Evolutionary conservation:
  ○ Microtubules and their aromatic residue patterns are highly conserved across eukaryotes (Janke and Bulinski, 2011; Hammond et al., 2008). *Euplotes* diverged from metazoans more than a billion years ago (Larson et al., 2022), suggesting that microtubule-based computation predates neurons and may be as ancient as other quantum-enabled biological systems such as chlorophyll and heme (Mitchell, 1961; Archibald, 2015).

We emphasize that only some links have direct quantum measurements (Wang, Reiter, Rodríguez-Rubio, Babcock). Others (Larson) establish function and structural dependence, making the hypothesis that quantum coherence contributes to microtubule function scientifically meaningful and falsifiable rather than speculative. The missing piece is a direct measurement of decoherence times in intact microtubules (Section 5).

#### 4.1.1 A quantitative estimate and a falsifiable working hypothesis

The mechanistic chain above is descriptive; here we make it deductive by combining the measured tryptophan geometry (Section 3.3.5) with Wang’s measured dielectric enhancement (Section 3.1) to derive an order-of-magnitude bound on the exciton decoherence time and a falsifiable scale law. The structural input is original to this work: the tryptophan nearest-neighbor spacings (∼1.3–1.8 nm) were obtained directly from deposited coordinates (PDB 1JFF, 3JAK), and they ground the estimate that follows.

**Screening-limited decoherence time**. The static-Coulomb contribution to electronic decoherence scales as Γ ∝ 1/ε^2^: the decoherence rate scales as the square of the system–environment coupling, and that coupling scales as 1/ε (Section 3.1.5). Tegmark’s most favorable estimate for microtubule electronic states, τ_T ≈ 10^−13^ s, assumes approximately bulk-water screening. To the extent that the dominant decoherence channel scales as 1/ε^2^, replacing the bulk dielectric constant with the confined-water value Wang measured in the 1–2 nm regime suppresses the rate by

$${\left(\varepsilon ∥,\text{confined}\;/\;\varepsilon ∥,\text{bulk}\right)}^{2}\approx {\left(1,030\;/\;74\right)}^{2}\approx 194,$$

extending the estimate to

$$\tau \approx \tau \_T\times {\left(\varepsilon ∥,\text{confined}\;/\;\varepsilon ∥,\text{bulk}\right)}^{2}\approx 10^{-13}\;s\times 194\approx 2\times 10^{-11}\;s.$$

Both dielectric values are Wang’s in-plane ε∥ from the same SDM measurement (bulk-like 74 above 4 nm; 1,030 ± 350 at h ≈ 1.5 nm), so the ratio is internally consistent. This is an order-of-magnitude bound for a *single* decoherence channel, and it rests on one load-bearing assumption: that the in-plane dielectric enhancement Wang measured for water in a flat 2D nanochannel applies to water in the tryptophan microenvironment of the microtubule lattice. That assumption is not established; it is precisely the transfer that Prediction 1 (scanning dielectric microscopy on purified microtubules, Section 4.5.1) is designed to test, and the estimate should be read as conditional on it.

**A falsifiable scale law (the dimensional-confinement hypothesis, H1)**. Wang’s ε∥(h) is non-monotonic: it peaks near h ≈ 1–1.5 nm and decays toward the bulk value above ∼4 nm. Because the screening-limited protection time scales as ε∥^2^ (τ ∝ ε^2^, since the decoherence rate Γ ∝ 1/ε^2^; Section 3.1.5), we predict:

**Working hypothesis (H1, the dimensional-confinement hypothesis)**. The tryptophan-exciton coherence-protection time τ(h) is governed by ε∥(h)^2^, and is therefore *maximized* near h ≈ 1–1.5 nm and *decreases* toward the bulk value above ∼4 nm.

**Null hypothesis (H0)**. τ is independent of confinement width, hydration state, and local dielectric response; any observed optical or electronic signatures arise from ordinary local photophysics with no confinement-dependent coherence protection.

H1 is falsified (and H0 retained) if direct measurements (two-dimensional electronic spectroscopy [2DES] coherence lifetimes or dielectric mapping; Predictions 5–6) show coherence times that are flat, increase monotonically with confinement width across the 1–4 nm range, or show no dependence on confinement.

We deliberately do not adopt a monotonic L^-2^ form: the measured dielectric response *peaks* rather than scaling as a power of confinement width, and the honest prediction is a maximum within the 1–2 nm window. Across the 1–4 nm range the present law and a monotonic L^-2^ form agree qualitatively (both predict stronger protection as confinement tightens), diverging only below the ∼1–1.5 nm peak, where Wang’s measured ε∥(h) turns over. This converts the dimensional-convergence observation from a description into a testable physical law.

**Relation to other timescale estimates**. The ∼10^−11^ s figure is deliberately conservative and belongs to a hierarchy of estimates that measure different things: Tegmark’s ∼10^−13^ s (most favorable, bulk-screening assumption); the present ∼10^−11^ s (a single static-Coulomb channel, using Wang’s measured confined-water ε); Hagan et al.‘s 10^−6^–10^−4^ s (a fuller treatment with additional corrections); and the ∼10^−8^ s effective coherence time obtained by reading Kalra et al.‘s measured ∼6.6 nm energy-migration range within the Orch OR framework (an empirical migration timescale whose interpretation as electronic coherence is model-dependent). These are not in conflict; they correspond to different decoherence channels, different levels of treatment, and, in Kalra’s case, a different observable (energy migration versus electronic coherence). Operationally, the predicted coherence would appear in 2DES as oscillatory cross-peaks persisting to tens of picoseconds, distinguishable by their phase signature from incoherent energy transfer (Prediction 6, Section 4.5.6): the measurable decomposition of a microtubule optical signal into a coherent (quantum) component.

### 4.2 Experimental limitations and open questions

#### 4.2.1 Transferability of Wang’s hBN results to protein surfaces

The single most important untested assumption in this synthesis is the transfer of Wang’s hBN-confined-water result to the protein context. Wang’s measurements were performed on water confined between hBN and graphite, not biological proteins, and both the confinement geometry and the surface chemistry differ. The relevant differences are concrete: hBN presents an atomically flat, chemically inert, weakly polar surface, whereas tubulin interfaces are topographically rough and chemically heterogeneous, carrying charged and polar groups (acidic C-terminal tails, exposed Glu/Asp/Lys side chains) and dense hydrogen-bonding sites. These differences could cut either way. If the anomalous in-plane ε∥ arises primarily from the geometric ordering of confined-water dipoles set by the channel width, it should transfer to any sufficiently narrow cleft; if instead it depends on the weak water–surface coupling specific to hBN, then strong electrostatic pinning and hydrogen bonding at a protein surface could suppress the cooperative dipolar response and lower ε∥. It therefore remains to be determined whether water confined between protein interfaces (e.g., tubulin protofilament clefts) exhibits comparably high ε∥ and σ∥. This is not a peripheral caveat but the load-bearing “if” of the proposed protection mechanism, and it is directly testable: scanning dielectric microscopy on purified microtubules (Prediction 1, Section 4.5.1) measures exactly this transfer, turning the synthesis’s chief vulnerability into its most decisive experiment.

#### 4.2.2 Protein dynamics and coherence

Synthetic porphyrin nanobelts are rigid, whereas proteins display thermal fluctuations. The difference is not merely mechanical but electronic: the nanobelts’ ring currents flow through a covalently locked, continuously conjugated circuit, so they demonstrate room-temperature π-delocalization *within a single rigid molecule*, not delocalization across a network of discrete, independently fluctuating aromatic side chains. The tryptophan case is the harder one, because coherence must survive both thermal motion and the geometric disorder of separate indole units; the nanobelt result establishes that nanometer-scale aromatic circuits can sustain room-temperature ring currents, not that a fluctuating Trp network will. Babcock et al.‘s robustness analyses speak directly to this gap: cooperative effects in extended networks are predicted to tolerate static and dynamic disorder up to ∼1000 cm^-1^, beyond typical biological vibrational energies, though this is a theoretical robustness bound, not a measurement of surviving coherence in a real Trp network. Neuronal microtubules in cortical pyramidal neurons are heavily stabilized through post-translational modifications and microtubule-associated proteins (Hammond et al., 2008; Baas et al., 2016; Kapitein and Hoogenraad, 2015), suggesting that biology actively maintains lattice order in precisely those cells where higher-order processing is thought to occur.

#### 4.2.3 Nanoconfined water: shield or noise source?

Superionic proton conductivity in nanoconfined water may introduce additional fluctuations, potentially contributing to decoherence rather than suppressing it. The key question is timescale: if proton dynamics occur on timescales slower than relevant electronic processes, effective averaging may render them less detrimental. Deciding whether nanoconfined water is net protective or disruptive requires direct decoherence-time measurements and noise spectroscopy rather than inference.

### 4.3 Revisiting the “too warm, too wet” objection

Tegmark’s 2000 analysis (Tegmark, 2000) predicted decoherence times of order 10^−13^ s for microtubule-based quantum states, based on bulk-water dielectric properties, point charges rather than dipoles, and neglect of structured environments. Hagan et al. (2002) corrected several key assumptions and predicted decoherence times of 10^−6^–10^−4^ s, but at the time this remained a theoretical debate.

The experimental results synthesized here speak directly to the three pillars of the objection:

- Too warm:
  (Rodríguez-Rubio et al., 2025) and (Babcock et al., 2024) both demonstrate room-temperature aromatic quantum phenomena at 298 K. Babcock’s networks maintain cooperative behavior even when modeled disorder exceeds kT by a factor of several, and photosynthetic systems provide a contested but influential precedent for room-temperature aromatic coherence (Engel et al., 2007; Collini et al., 2010; Lorenzoni et al., 2025). Temperature alone is insufficient to preclude coherence.
- Too wet:
  (Wang et al., 2025) and (Reiter et al., 2016) show that water at 1–2 nm confinement is not “bulk water in a smaller box” but a distinct phase with dramatically altered dielectric and transport properties, including direct evidence of quantum coherence in the case of Reiter. (Hagan et al., 2002) had predicted that interfacial water would differ qualitatively from bulk; the nanoconfined water data provide experimental support for this key premise.
- Too noisy/complex:
  Protein dynamics and environmental complexity are often invoked as additional decoherence sources. Yet Babcock’s disorder robustness, the structural stabilization of neuronal microtubules (Baas et al., 2016; Kapitein and Hoogenraad, 2015), and the cooperative nature of extended networks all argue against a simple “more complexity ⇒ no coherence” narrative. Larger networks can be more robust due to cooperative effects, and biology invests biochemical resources in maintaining microtubule order in neurons, which is at least consistent with the idea that such order matters functionally.

These observations do not prove long-lived quantum coherence in microtubules, but they substantially weaken the original environmental objections and justify direct measurement.

A more speculative possibility, that phase coupling across multiple frequency domains (kHz → PHz) could relax the requirement for long-lived coherence at any single frequency, has been proposed by Hameroff et al. (2026) and is discussed separately in the Supplementary Material. We note it here only for completeness: the argument of this section rests on the screening and cooperative-network considerations above and does not depend on the time-crystal model.

### 4.4 Implications for consciousness theories and anesthesia

Orch OR (Hameroff and Penrose, 1996; Hameroff and Penrose, 2014) specifically proposed that tubulin aromatic networks in microtubules could support quantum-coherent processes contributing to consciousness. Several pieces of independent evidence now align with the substrate-level assumptions of this theory:

- Extended aromatic π-networks are demonstrably capable of coherence at 298 K (Rodríguez-Rubio et al., 2025).
- Microtubule tryptophan networks show cooperative optical behavior at 298 K (Babcock et al., 2024).
- Nanoconfined water at microtubule-relevant lengthscales exhibits anomalous dielectric properties and quantum behavior (Wang et al., 2025; Reiter et al., 2016).
- Microtubule structural integrity is functionally critical for cellular computation in *Euplotes* (Larson et al., 2022).

Craddock et al. (2015) added a further, clinically relevant piece: diverse general anesthetics bind preferentially to tubulin hydrophobic pockets rich in tryptophan. These are the exact aromatic environments in which Babcock observed superradiant-like behavior. This motivates a specific experimental test linking quantum coherence to consciousness-related pharmacology:

Prediction: Clinically relevant concentrations of general anesthetics should reduce or disrupt the cooperative fluorescence signatures measured by Babcock et al. in microtubule tryptophan networks, in a dose-dependent and reversible manner (Section 4.5.3).

If such an effect were observed and correlated with anesthetic potency, it would support the hypothesis that anesthetics act, at least in part, by disrupting collective aromatic quantum states. If no such effect is observed, receptor-centric models of anesthesia (Franks and Lieb, 1994; Hemmings et al., 2005; Campagna et al., 2003) remain sufficient, and the quantum contribution is either absent or negligible.

Thus, while a direct link between microtubule quantum coherence and consciousness remains unproven, the convergent evidence makes it a well-posed empirical question rather than a purely speculative one.

Preliminary experimental evidence:

Prediction 3 (Section 4.5.3) has received preliminary support. Kalra et al. (2023) measured exciton migration over ∼6.6 nm in microtubules at ambient temperature using ultrafast spectroscopy, with excited-state lifetimes on the order of nanoseconds, and found that the migration was reduced by clinical anesthetics (isoflurane and etomidate) at pharmacologically relevant concentrations. As set out in the timescale hierarchy of Section 4.1, whether this ∼6.6 nm migration reflects sustained electronic coherence or incoherent energy transfer is model-dependent and remains the open question that Prediction 6 is designed to resolve. With that caveat, the result is consistent with:

1. The presence of anesthetic-sensitive electronic excitations in microtubule aromatic networks at physiological temperature;
2. Interference by general anesthetics with those excitations, consistent with the Craddock binding model (Craddock et al., 2015); and
3. The aromatic networks identified by Babcock et al. (2024) as plausible substrates for the anesthetic-sensitive states probed by Kalra.

These observations do not establish that sustained quantum coherence exists in microtubules, let alone that it causes consciousness; they establish that the quantum-substrate hypothesis is empirically testable and that the first available tests are consistent with, rather than refuting, its predictions. Further work should focus on direct correlation between anesthetic potency, aromatic-network perturbation (Prediction 3, Section 4.5.3), and decoherence-time measurement (Prediction 6, Section 4.5.6).

### 4.5 Testable predictions and future experiments

Below we summarize key falsifiable predictions motivated by the mechanistic chain. Each follows a simple structure: hypothesis, experiment, expected outcome, and falsification criterion.

#### 4.5.1 Prediction 1: dielectric properties of microtubule nanoconfinement

- Hypothesis: Water confined between microtubule protofilaments exhibits ε∥ ≫ 80 (ideally ε∥ ≥ 500) at 1–2 nm separations.
- Experiment: Use SDM on purified, stabilized microtubules to map in-plane dielectric response as a function of local confinement geometry and hydration.
- Expectation: A pronounced ε∥ peak near 1–2 nm, qualitatively similar to Wang’s hBN channels; possible differences in magnitude due to surface chemistry.
- Falsification: ε∥ remains bulk-like (∼80) across all biologically relevant confinement lengths.

#### 4.5.2 Prediction 2: NMR detection of aromatic delocalization in tubulin

- Hypothesis: Assembly into microtubules generates ring-current-like chemical shift patterns in selected tryptophan residues.
- Experiment: ^1^H/^15^N NMR of labeled tubulin across free Trp, monomer, dimer, and assembled microtubule states.
- Expectation: Systematic inner/outer shielding pattern and significant chemical shift changes (>0.5 ppm) between dimers and assembled microtubules.
- Falsification: No state-dependent chemical shift changes beyond local environmental effects.

#### 4.5.3 Prediction 3: anesthetic disruption of microtubule superradiance

- Hypothesis: General anesthetics reduce cooperative emission from microtubule tryptophan networks by perturbing aromatic-network geometry and transition-dipole coupling.
- Experiment: Repeat Babcock’s quantum yield measurements in the presence of clinically relevant concentrations of isoflurane, propofol, and xenon; include fluorescence lifetime and polarization controls.
- Expectation: QY and/or radiative rate reductions at anesthetic concentrations, with dose-dependence and reversibility upon washout.
- Falsification: No measurable change in QY or lifetimes at clinically relevant doses.

#### 4.5.4 Prediction 4: aromatic residue requirement

- Hypothesis: Specific tryptophan residues are necessary for observed cooperative optical effects.
- Experiment: Systematic Trp→Ala or Trp→Phe mutagenesis in tubulin, expression, and assembly into microtubules followed by QY measurements.
- Expectation: Loss of superradiant-like enhancement when key interfacial Trp residues are mutated; partial reductions for conservative aromatic substitutions.
- Falsification: QY and scaling remain unchanged across mutations.

#### 4.5.5 Prediction 5: solvent and hydration dependence

- Hypothesis: Cooperative optical behavior depends critically on structured interfacial/nanoconfined water (the wall-adjacent and inter-protofilament shell), rather than on bulk luminal water.
- Experiment: QY and lifetime measurements under controlled dehydration, solvent substitution (e.g., low-ε solvents), and D_2_O vs. H_2_O.
- Expectation: QY maximized near physiological hydration in water; diminished under dehydration or low-ε organic solvents.
- Falsification: Cooperative signatures are insensitive to hydration state and solvent dielectric.

#### 4.5.6 Prediction 6: direct decoherence-time measurements

- Hypothesis: Microtubule aromatic networks support electronic coherence on timescales longer than Tegmark’s 10^−13^ s estimate, potentially approaching or exceeding picoseconds.
- Experiment: Two-dimensional electronic spectroscopy (2DES) (Jonas, 2003) on purified, stabilized microtubules (and mutants) to measure coherence lifetimes and noise spectra.
- Expectation: Coherence signals on fs–ps timescales, with sensitivity to structural perturbations and anesthetics.
- Falsification: No measurable coherence beyond instrument noise, or lifetimes consistently limited to <10^−13^ s.

### 4.6 From nanoscale signatures to cellular recordings

Cellular-scale recordings should not be interpreted as direct quantum measurements. A quantum contribution would be inferred only if a nanoscale optical or electrodynamic signature survives a hierarchy of controls drawn from the predictions above: dependence on microtubule assembly and aromatic-network integrity, including loss under tryptophan mutation or aromatic substitution (Prediction 4); hydration and isotope dependence (Prediction 5); reversible anesthetic dose–response with washout (Prediction 3); and correlation with 2DES phase-coherent cross-peaks or fluorescence-lifetime changes (Prediction 6). On this logic, a cellular observable would be decomposed into a classical structural component, a local photophysical component, and a candidate coherent component defined operationally by phase persistence, perturbation specificity, and washout reversibility. It is this decomposition under controls, rather than any single recording, that would distinguish a genuine quantum signature from classical photophysics.

## 5 Conclusion and future directions

### 5.1 Summary of convergent evidence

We have synthesized experimental results from five independent research programs (across materials physics, synthetic chemistry, biophysics, and cell biology) to argue that the question of microtubule quantum coherence is now primarily empirical, not conceptual:

- Nanoconfined water at 1–2 nm exhibits anomalously high dielectric constants and quantum-coherent proton motion at 295–300 K (Wang et al., 2025; Reiter et al., 2016).
- Aromatic π-networks can maintain extended delocalization at 298 K in 2–3 nm porphyrin rings (Rodríguez-Rubio et al., 2025).
- Microtubule tryptophan networks exhibit size-dependent optical behavior at 298 K consistent with cooperative quantum effects and robust to substantial disorder (Babcock et al., 2024).
- Microtubule structural integrity is functionally critical for cellular computation in unicellular organisms, with stabilization enhancing and depolymerization degrading performance (Larson et al., 2022).

The four experimental prerequisites for biologically relevant quantum coherence (appropriate environment, capable substrate, empirical biological signatures, and functional dependence) are each supported by at least one independent study; the fifth element of the mechanistic chain, evolutionary conservation, is a plausibility consideration rather than an experimental prerequisite. What remains unmeasured is the key parameter that would settle the debate: the decoherence time of putative quantum states in intact microtubules.

### 5.2 From “whether” to “what role”

The data synthesized here suggest that the long-standing “too warm, too wet” objection to quantum biology has been significantly weakened. Quantum coherence in biological aromatic systems at physiological temperature is now a serious, empirically supported possibility in multiple contexts (porphyrin belts at 298 K, microtubule tryptophan networks), and a contested one in others (photosynthesis). The relevant question is therefore shifting from whether quantum coherence can exist in neural cytoskeletal structures to what functional roles, if any, such coherence plays.

Multiple possibilities exist: enhanced energy transfer, improved sensitivity to weak fields or signals, long-range intra- and intercellular communication, or a contribution to the integrative processes underlying consciousness as posited by Orch OR (Hameroff and Penrose, 1996; Hameroff and Penrose, 2014). Larson’s demonstration of microtubule-based finite-state-machine behavior in *Euplotes* suggests that microtubules can implement non-trivial computation independently of neurons; neuronal microtubules might build on this ancient machinery in more complex ways.

Decisive experiments, especially direct decoherence-time measurements and anesthetic perturbation studies, can now be formulated and prioritized. If decoherence times are found to be extremely short (<10^−13^ s), functional quantum roles in microtubules are effectively ruled out. If, on the other hand, coherence persists on timescales comparable to or longer than those already observed in other biological systems, new avenues open for integrating quantum processes into our models of cellular and neural function.

In either outcome, the path forward is clear: measure decoherence times in microtubules under biologically relevant conditions. The convergent evidence summarized here provides a strong justification for investing in that measurement.

## Data Availability

All data analyzed in this study are available in the published literature cited in the References section. Scripts used to generate figures are available from the corresponding author upon reasonable request.
