# Supplementary material for: Dimensional convergence at the nanoscale: a novel synthesis of experimental evidence justifying direct measurement of quantum coherence in microtubules
Source: Front Mol Biosci. 2026 Oct 9;13:1840116. doi: 10.3389/fmolb.2026.1840116 (PMC13690665; doi:10.3389/fmolb.2026.1840116)
Supplement: Supplementary file 1 [file Supplementaryfile1.docx]

# Supplementary Material

**Article:** Dimensional Convergence at the Nanoscale: A Novel Synthesis of Experimental Evidence Justifying Direct Measurement of Quantum Coherence in Microtubules

**Author:** Justin Echternach

*Note: Bracketed reference numbers below (e.g., [41]) refer to the reference list of the main article.*

# Supplementary Appendix A — A Speculative Interpretive Note

*The following framework is included for completeness and is explicitly speculative. It is separable from, and not required by, the central argument of this paper: the dimensional co-localization at the 1–2 nm scale (Sections 2–3) and the screening-limited decoherence estimate and the Dimensional-Confinement Hypothesis (Section 4.1). The fractal time-crystal model of Hameroff et al. (2026) has not been experimentally validated by its own authors; the frequency mapping below is presented as one possible interpretive organization, not as evidence for the present claims. Readers focused on the falsifiable core may skip this appendix.*

## A.1 Multi-Frequency Spatiotemporal Framework (Hameroff Time-Crystal Model)

The five independent lines of evidence described above establish **spatial convergence** at the 1–2 nm scale as a necessary condition for quantum coherence in microtubules. Recent theoretical work by Hameroff et al. (2026) [41] extends this picture by proposing that microtubules operate as **fractal time crystals** supporting phase-coupled oscillations across multiple frequency domains spanning twelve orders of magnitude (kHz → PHz). Placed against Hameroff’s proposed frequency subsystems, the experimental evidence synthesized here can be organized as shown in Table A1. We present this as a framework *proposed by Hameroff et al.*, against which the present evidence can be arranged, rather than as an independent derivation: the mapping is suggestive but interpretive, and indicates, without establishing, that dimensional co-localization at the nanoscale could supply a **spatial organizing principle** for the **temporal hierarchy** Hameroff et al. propose.

**Table A1: Frequency Mapping Between Experimental Evidence and Time Crystal Subsystems**

| Experimental Evidence | Hameroff Subsystem | Frequency Domain | Physical Mechanism |
| --- | --- | --- | --- |
| Wang/Reiter (exterior water + C-termini) | Exterior water layer + ionic coupling | **kHz** | Surface screening, dipolar relaxation |
| Wang/Reiter (core water channel) | Hollow core + inter-protofilament clefts | **GHz** | Quantum proton motion in confined geometry |
| Saxena/Bandyopadhyay (2020, 2023) | Lattice phonons | **MHz (~10 MHz)** | Optimal frequency for Orch OR; coherent vibrations |
| Rodríguez-Rubio (π-electron transitions) | Aromatic ring currents | **THz** | Delocalized electron dynamics at 298 K |
| Babcock (UV emission, 330 nm) | Cooperative photon emission | **PHz (~750–1000 THz)** | Superradiant UV fluorescence |
| Babcock (aromatic↔collective coupling) | Cross-frequency coupling | **THz–MHz** | Bridge between electronic substrate and collective modes |
| Larson (gait coordination in *Euplotes*) | Functional validation | **Hz (behavioral)** | Macroscopic output from multi-frequency computation |
| **Dimensional convergence (all studies)** | **Spatial anchor** | **1–2 nm** | **All frequency subsystems co-localize at this scale** |

Hameroff’s model proposes that microtubules support a **Fibonacci topological geometry** (helical lattice with Fibonacci winding numbers 3, 5, 8) that organizes oscillations across this twelve-order-of-magnitude frequency range [41]. Multi-frequency operation is presented not as a weakness but as a **feature** that addresses a central objection to microtubule-based quantum processing. Rather than requiring coherence times spanning milliseconds to seconds (the original Tegmark critique [1,2]), the time crystal framework allows **short-lived coherence at high frequencies** (e.g., THz aromatic ring currents, GHz proton motion) to couple **via phase relationships** to slower collective modes (MHz lattice phonons, kHz dipolar oscillations), ultimately producing behaviorally relevant outputs at Hz frequencies (Larson’s gait coordination) [10].

**Observations that can be compared with the proposed multi-frequency framework:**

1. **Kalra et al. (2023)** [40] measured **exciton migration over ~6.6 nm** in microtubule lattices at ambient temperature, with excited-state lifetimes on the order of nanoseconds. Read within the Orch OR framework [2], this migration has been interpreted as an effective coherence time of order 10⁻⁸ s; as noted in the timescale hierarchy of Section 4.1, however, whether it reflects sustained electronic coherence or incoherent energy transfer is model-dependent. The migration was also **reduced by clinical anesthetics** (isoflurane and etomidate), consistent with Prediction 3 (Section 4.5) that anesthetic binding perturbs these aromatic networks.
2. **Bandyopadhyay group (Saxena et al. 2020, 2023)** [29-31] identified **ballistic conductance** in brain microtubules with resonant behavior near **10 MHz**, precisely the frequency Hameroff proposes for optimal Orch OR coupling between electronic (THz) and phononic (MHz) subsystems.
3. **Babcock et al. (2024)** [9] demonstrated that UV emission (PHz) from tryptophan networks couples to **collective optical behavior** consistent with superradiance, a **cross-frequency phenomenon** where high-frequency photon emission reflects lower-frequency coherent phase alignment among aromatic dipoles (THz–MHz range). Hameroff et al. (2026) explicitly cite Babcock’s findings as evidence for cooperative dynamics in microtubule aromatic networks [41].
4. **Larson et al. (2022)** [10] showed that microtubule structural integrity enables **gait coordination** in *Euplotes* (behavioral timescale: Hz), with paclitaxel stabilization enhancing performance and nocodazole depolymerization abolishing it. This is compatible with, but does not demonstrate, the possibility that microtubule-scale processes couple to slower cellular outputs.

**Spatiotemporal synthesis:**

The convergence documented here is, in the first instance, **spatial**: nanoconfined water (Wang, Reiter), aromatic networks (Rodríguez-Rubio, Babcock), and functional microtubule architecture (Larson, Sahu, Saxena) co-localize at the 1–2 nm scale, providing a common geometric substrate. Whether this spatial convergence also underwrites a *temporal* organization (for example, the multi-frequency, time-crystal-style coupling explored speculatively in Appendix A) is a separate and unproven question that the present synthesis neither requires nor establishes. What the synthesis does establish is that the spatial conditions are met and that the decisive test is now experimental: direct measurement of decoherence times in intact microtubules.

The questions pursued here also bear on domains beyond fundamental biophysics: microtubules are long-established targets in oncology and anesthesiology, their chiral nanoscale architecture is of growing interest in bio-inspired materials, and the spectroscopic signatures discussed here may in time inform imaging and characterization methods. These applied directions depend, however, on first resolving the collective-versus-classical question this synthesis addresses, and are noted as motivation for future work rather than as claims the present evidence can substantiate.
